# Enhancing anticipation control of the posture system in the elderly wearing stroboscopic glasses

**DOI:** 10.1186/s12984-025-01549-4

**Published:** 2025-05-05

**Authors:** Yi-Ching Chen, Yi-Ying Tsai, Yen-Ting Lin, Ing-Shiou Hwang

**Affiliations:** 1 Department of Physical Therapy, College of Medicine, National Cheng Kung University, Tainan City, Taiwan; 2 Department of Physical Therapy, College of Medical Science and Technology, Chung Shan Medical University, Taichung City, Taiwan; 3 Physical Therapy Room, Chung Shan Medical University Hospital, Taichung City, Taiwan; 4 Department of Ball Sport, National Taiwan University of Sport, Taichung City, Taiwan; 5 Institute of Allied Health Sciences, College of Medicine, National Cheng Kung University, Tainan City, Taiwan

**Keywords:** Stroboscopic vision, Phase-amplitude coupling, EEG, Posture, Open-looped control

## Abstract

**Background:**

Stroboscopic vision (SV), known for providing intermittent visual input, has been recently integrated into postural training to improve proprioceptive awareness. This research examined the impact of SV on cortico-posture coupling in older adults, along with the related changes in postural control throughout a spectrum of feedback and feedforward processes.

**Methods:**

A total of thirty-three adults, averaging 66.1 ± 2.5 years of age, were tasked with maintaining an upright posture on a stabilometer, utilizing either complete or intermittent visual guidance. Stabilogram diffusion analysis (SDA) was employed to assess balance strategies based on postural sway, while phase-amplitude coupling (PAC) between postural fluctuations and scalp EEG provided insights into the associated neural control mechanisms.

**Results:**

SV resulted in significantly increased postural sway as compared with that of full-vision feedback (*p* < 0.001). SDA results indicated greater critical point displacement (CD) (*p* < 0.001), short-term diffusion coefficients (Ds) (*p* < 0.001), and scaling exponents (Hs) (*p* = 0.014) under SV conditions. PAC analysis revealed that the coupling between the postural fluctuation phase and cortical oscillation amplitude in the theta and alpha bands of the fronto-central area was significantly greater in the SV condition than in the full-vision condition (*p* < 0.001). Additionally, SV led to increased beta PAC in the frontal and sensorimotor areas compared to that of full vision (*p* < 0.001), which negatively correlated to SV-dependent changes in open-loop gain (Hs) (*p* < 0.05).

**Conclusions:**

SV transitions postural sway towards an open-loop process and influences cortico-posture interactions in older adults, emphasizing a neuromotor adaptation to the uncertainty in feedforward predictions when utilizing intermittent visual feedback.

## Background

As individuals age, visual feedback assumes a more dominant role in maintaining static and dynamic balance [1, 2], compensating for the significant degenerative decline in the vestibular and proprioceptive systems [3–5]. However, visual processing is slower than that of non-visual channels due to the complexity of the visual pathway [6, 7], which involves multiple stages of image processing and multimodal sensory integration. Therefore, older adults, who rely on vision to solve sensory ambiguity, are more susceptible to falls during increased attentional demand, diminishing their ability to adapt to challenging situations such as walking with visual clutter, on uneven surfaces, and so on [8, 9]. To mitigate the risk of injuries, rehabilitative training aims to reduce visual reliance on postural control in older adults [10–12], individuals with musculoskeletal injuries [13, 14], and those with neurological disorders [15].

Using liquid crystal technology, stroboscopic glasses flicker between clear and opaque states, intermittently blocking visual inputs from the environment. Previous studies have shown that combining sports skills training with stroboscopic vision (SV) can improve reaction time and hand-eye coordination, optimizing sport performance in visually challenging conditions [16–18]. SV may also facilitate sensory reweighting by causing greater reliance on non-visual inputs. Consequently, postural training combined with SV is presumed to enhance proprioceptive awareness and improve sensory integration [19, 20]. For movements relying on internal body awareness, such as posture adjustments or blindfolded reaching, SV may improve the accurate estimation of internal body states within the egocentric reference frame by enhanced proprioceptive inputs [21]. This allows for efficient reflexive postural adjustments to counteract destabilization over time in the absence of visual feedback [22]. According to the constrained action hypothesis, intermittent disruptions in the visual-perception-action loop can redirect attention from visuospatial focus to movement patterns during execution [23, 24]. If SV indeed improves open-loop control during unsteady stances by leveraging proprioceptive information, its training benefits could be particularly valuable for older adults, who are at a higher risk of falls.

Multiple lines of evidence have shown that SV induces cortical reorganization for movement execution in humans. For instance, Hülsdünker et al. (2023) found that SV delayed visual motion perception in a computer-based reaction test, resulting in prolonged N2 latency and reduced N2 amplitude in the motion-sensitive visual area [25]. After a 10-week SV training intervention, reaction time to visual motion stimuli significantly improved, with a corresponding decrease in stimulus-locked N2 latency indicating faster visual information processing [17]. Additionally, postural training with SV on an unsteady surface resulted in widespread plastic changes in EEG relative power and EEG-EEG connectivity across different sub-bands, supporting the deactivation of the dorsal visual stream and visual error processing [26]. Despite these findings, no research has directly supported brain–posture interaction by specifically linking the variations in the roles of open-loop and closed-loop control over postural regulation with the application of SV.

In addition to postural fluctuation dynamics, this study investigated phase-amplitude coupling (PAC) between low-frequency postural fluctuations (< 2 Hz) and high-frequency scalp EEG in various sub-bands (4–35 Hz) for older adults during stabilometer stance with and without SV. This novel approach provides additional insights into the effects of SV on cortico-posture coupling [27, 28], beyond what traditional coherence analysis offers, by quantifying the linear relationship between signals at a given frequency. The hypothesis of this study was that the topological distribution of PAC between postural fluctuations and EEG in the theta (4–7 Hz), alpha (8–12 Hz), and beta (13–35 Hz) bands would vary with SV application, favoring open-loop control over postural sway for older adults during stabilometer stance.

## Methods

### Ethical approval and participants

The study’s data partly included findings from a prior investigation conducted by Tsai et al. (2022), which explored the effects of stroboscopic vision at 3 Hz on regional activity and inter-regional connectivity during stabilometer stance. However, this study revealed novel aspects of the stroboscopic vision effect at 1 Hz, with a specific focus on comprehensive approaches such as stabilogram diffusion analysis (SDA) and phase–amplitude coupling (PAC) of postural fluctuations and scalp EEG [27, 28], which were not reported in the previous work. The study was approved by the authorized institutional human research review board at National Chung Cheng University Hospital (A-ER-107-099-T). Prior to the experiment, all subjects read and signed personal consent forms in accordance with the Declaration of Helsinki.

Thirty-three older adults over 60 years old (18 females and 15 males; age: 66.1 ± 2.5 years) participated in this study. Most participants were recreationally active with regular exercise habits. They had corrected-to-normal vision and no known cognitive problems, history of falls, or diagnoses of neurological and musculoskeletal disorders requiring medication, except for some with mild hypertension managed with regular medication.

### Experimental procedures

**Fig. 1:**
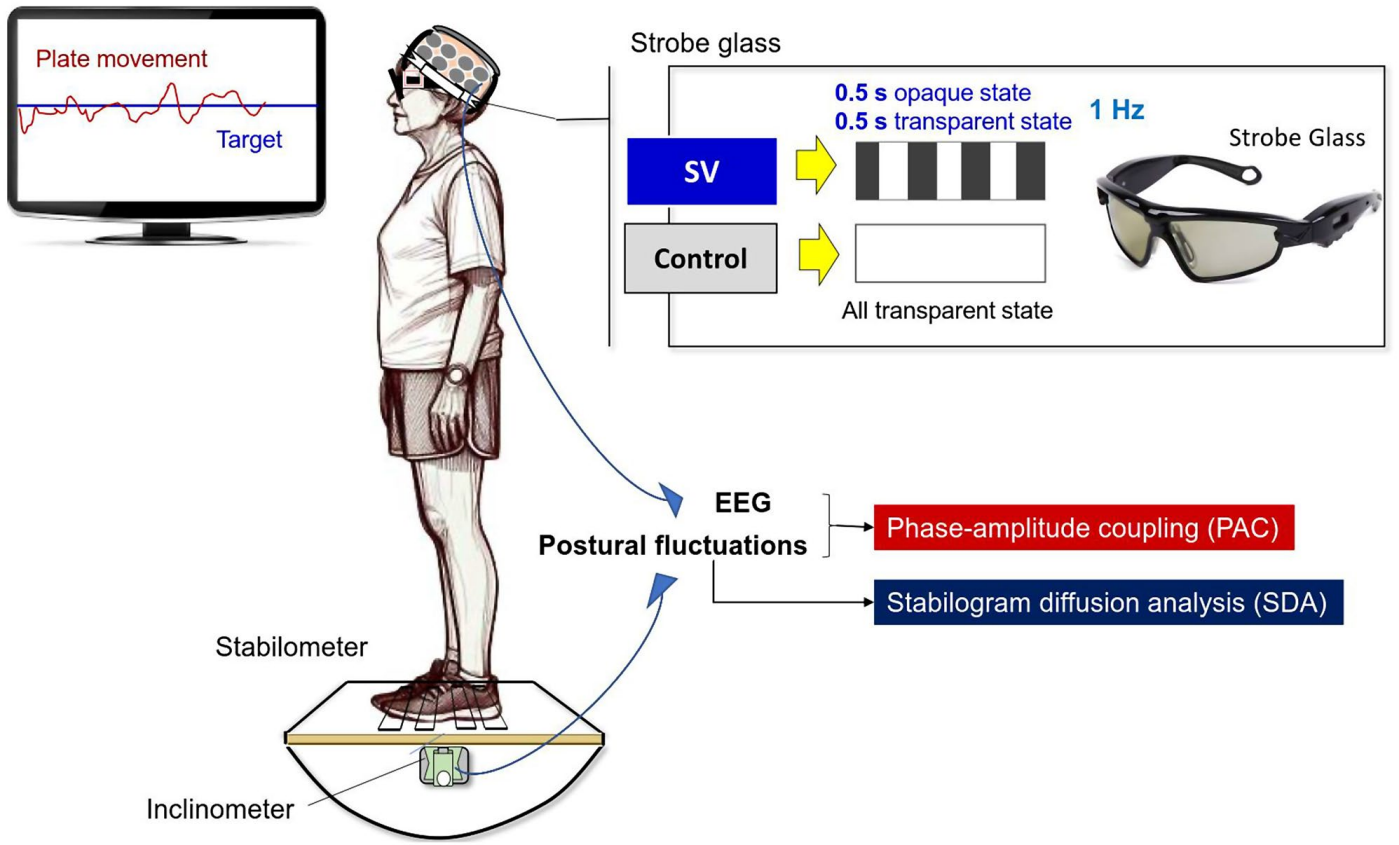
The experimental setup for measuring the dynamics of postural fluctuations and scalp EEG signals involves visually-guided stabilometer stance in both the control and stroboscopic vision (SV) conditions. In the SV condition, stroboscopic glasses intermittently block visual feedback of balance performance at 1 Hz while subjects regulate their upright stance on the stabilometer. In the control condition, subjects wear the glasses and visually track the stabilometer movement trajectory without visual occlusion. Phase-amplitude coupling analysis between the phase of postural fluctuations and scalp EEGs is conducted to characterize cortico-posture interaction during postural regulation. Stabilogram diffusion analysis (SDA) is used to analyze the non-linear dynamics of postural fluctuations

The postural task employed in this study was the stabilometer stance, a widely used method for enhancing stance stability among elderly individuals and neurological patients experiencing balance impairments. Participants stood barefoot on a wooden stabilometer measuring 50 cm × 58 cm (radius: 25 cm; height: 18.5 cm), which was partially surrounded by a custom-built wooden handrail. The stabilometer was capable of rotating along its sagittal axis, with a maximum roll excursion of 20°. Participants received instructions to keep the stabilometer level by using real-time visual feedback of its trajectory and a horizontal target line displayed on a computer monitor (Fig. 1). During the task, participants wore stroboscopic glasses (Visionup Athlete VA11-AF, Japan). In the full vision condition, the glasses allowed unobstructed visual feedback for maintaining balance. In the SV condition, the glasses alternated between opaque and transparent states for 0.5 s each at a frequency of 1 Hz, effectively halving the visual feedback exposure (Fig. 1). Each trial lasted for 45 s, and participants completed three experimental trials in both the SV and full-vision (control) conditions in alternating order. To mitigate fatigue, 3-minute rest intervals were provided between trials. Half of the participants began with an SV trial, while the other half commenced with a full vision trial.

### Instrumentation setting

Angular movements of the stabilometer were monitored using an inclinometer (Model FAS-A, LORD MicroStrain, USA) mounted on its rotational axis. These angular measurements were sampled at 1 kHz via an analog-to-digital converter (Model 6341, National Instruments, USA) and processed using the LabVIEW software (LabVIEW v8.5, National Instruments, USA). Concurrently, cortical activity during the stabilometer stance was recorded using a scalp EEG system equipped with Ag-AgCl electrodes and a 40-channel NuAmps amplifier (NeuroScan Inc., El Paso, USA), following the 10–20 electrode placement system. The ground electrode was situated along the midline, positioned anterior to Fz. For offline electrooculography (EOG) analysis, electrodes were placed at the outer canthi of both eyes and infra- and supra-orbitally at the right eye. All electrode impedances were maintained below 5 kΩ. The EEG data were captured with a band-pass filter set to 0.1–100 Hz and a sampling rate of 1 kHz.

### Data analysis

**Fig. 2:**
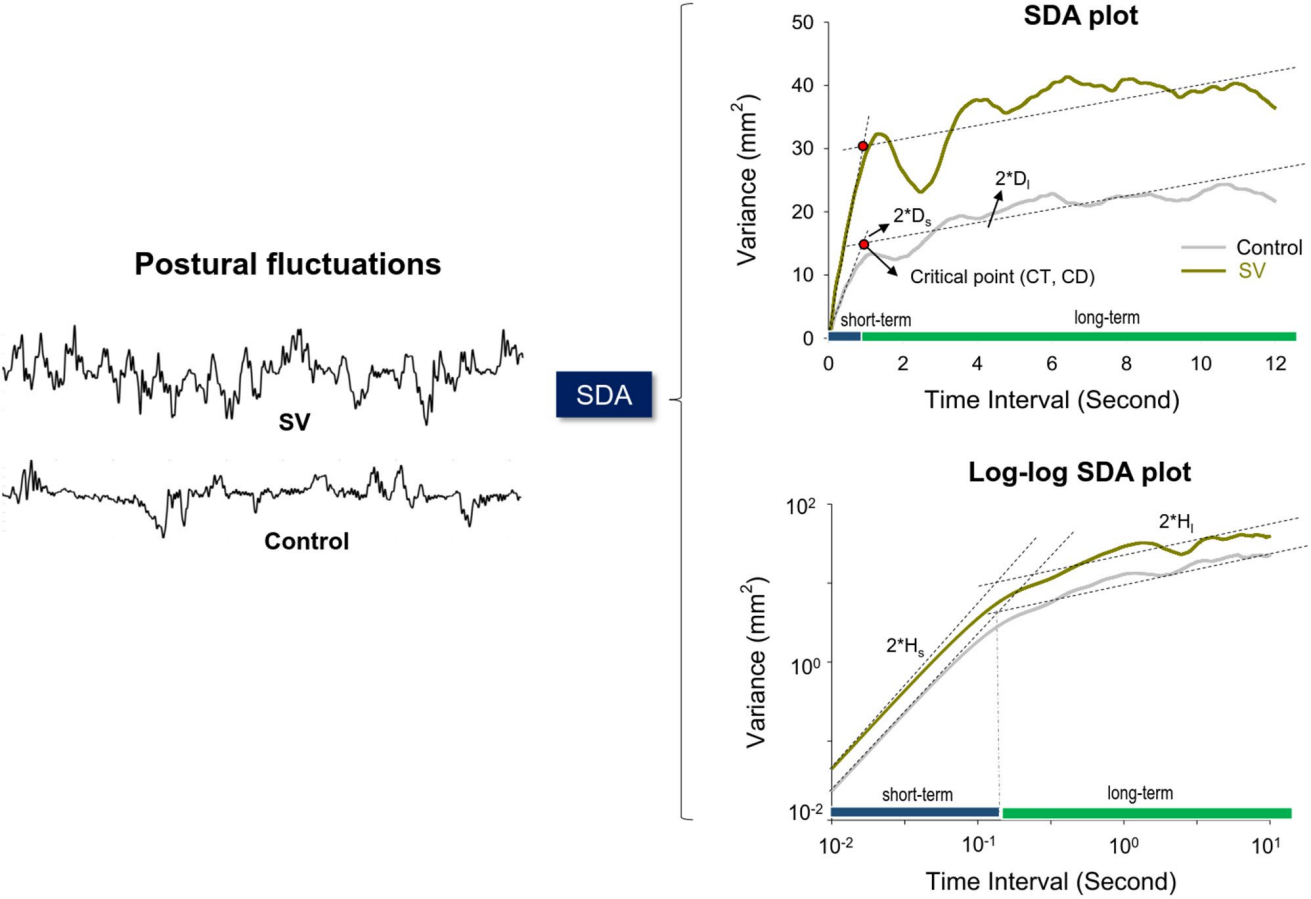
A typical example of linear and log–log stabilogram diffusion plots in the control and SV conditions. The diffusion plots can be modeled with a pair of regression lines that intersect at the critical point. The critical point of time (CT) and critical point of displacement (CD) denote the time-interval scale and variance scale of the critical point, respectively. The short-term and long-term regions are determined by CT, which correspond to open-loop and closed-loop control strategies for postural fluctuations, respectively. Stabilogram diffusion parameters (Ds, Dl, Hs, Hl) are determined by the slopes of lines fitted to the short-term or long-term regions

The angular stabilometer movements were conditioned using a 4th-order low-pass Butterworth filter with a cutoff frequency of 4 Hz. Postural fluctuations were defined as the filtered angular stabilometer movements after the removal of their linear trends. The size of the postural fluctuations was indexed with root mean square (RMS). Stabilogram diffusion analysis (SDA) was used to analyze postural sway dynamics [29, 30], which models the sway as a stochastic process to characterize the random and deterministic components of postural fluctuations. SDA describes the power-law relationship between the mean-squared value (or variance) of postural fluctuation time-series (< *dPF*^2^> ) and the time interval (*dt*) in which these values occur. It is formulated as $\left\langle {dP{F^2}} \right\rangle = \left\langle {{{[x(t + dt) - x(t)]}^2}} \right\rangle$, where <•> indicates the mean of the detrended postural fluctuation time-series. Twelve-second segments in postural fluctuation were repeated to compute <*dPF*^2^> with increasing dt values. The diffusion plot (linear–linear plots or log–log plots) was the mean square of the detrended time-series of the postural fluctuation data <*dPF*^*2*^> against the time intervals *dt* (Fig. 2). The diffusion plots can be modeled with two linear regression lines that intersect at the critical point, reflecting the transition from short-term to long-term postural sway processes [29, 30]. Critical time (CT) represents the time scale at the critical point, while critical displacement (CD) indicates variations in postural fluctuations (Fig. 2). The critical point also reflects a strategic shift in postural control [29–31]. A greater CD indicates a shift in postural control from predominant open-loop control to closed-loop control during larger postural sway. A greater CT reflects a longer lag time for the postural system to engage the closed-loop control mechanism. The slopes of the regression lines for the short-term and long-term regions in the linear–linear diffusion plot are the effective diffusion coefficients, Ds and Dl, respectively, which parameterize the control of the stochastic activities of postural fluctuations in their respective regions. The short-term and long-term scaling exponents (Hs and Hl) are derived from linear fits of the log–log plot of the SDA. An Hs greater than 0.5 indicates that the postural system in the short-term region is governed by the open-loop process, as the past and future data series are positively correlated, exhibiting persistence [29, 30]. Conversely, an Hl smaller than 0.5 suggests that the postural system in the long-term region is governed by the closed-loop process, with the past and future data series negatively correlated, demonstrating anti-persistence. The SDA variables were analyzed in MATLAB R2019a software (MathWorks, USA).

**Fig. 3:**
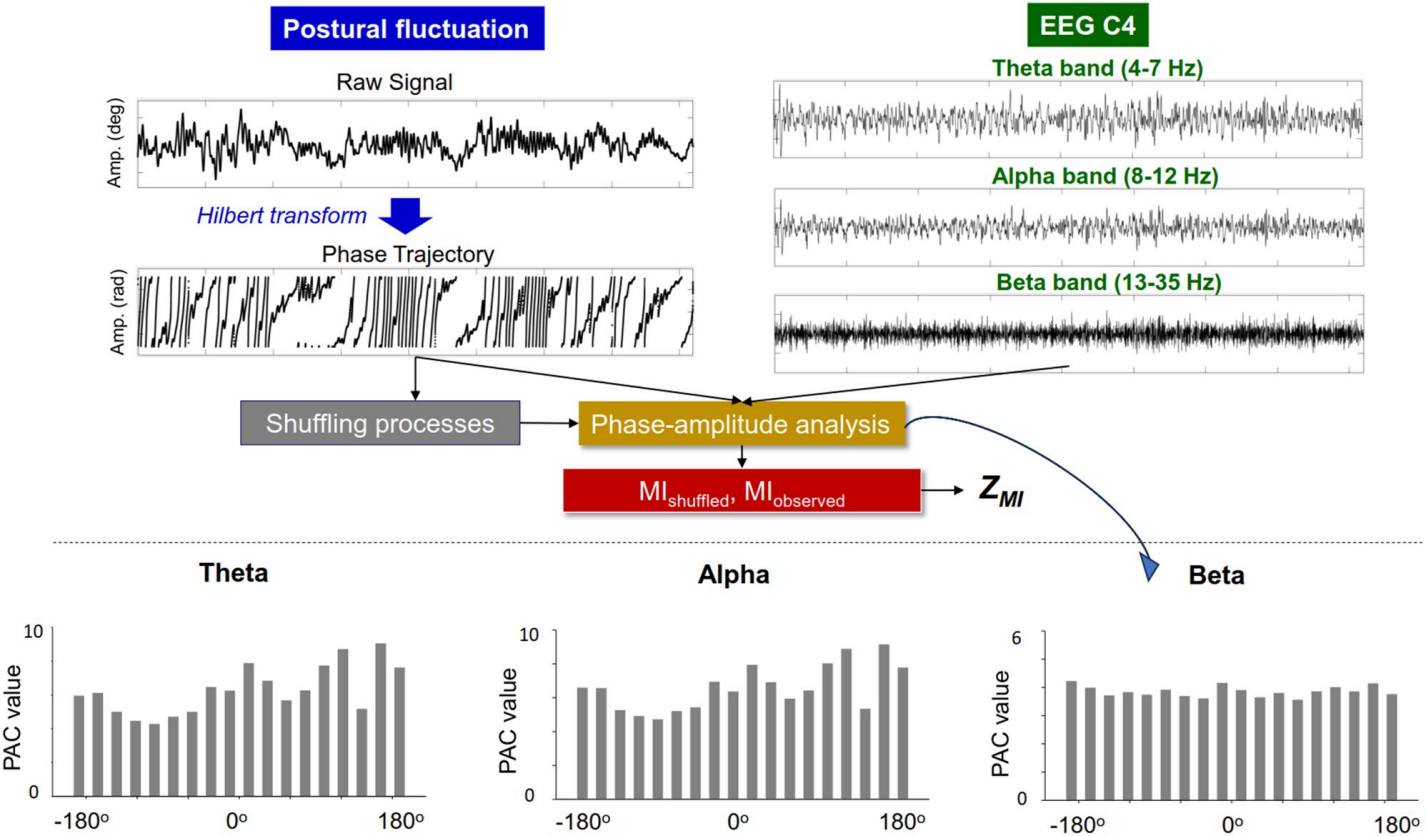
Procedure flowchart to calculate phase–amplitude coupling (PAC) between postural fluctuations and scalp EEG. The phase component of the postural fluctuations is extracted using the Hilbert transform. The scalp EEG at the C4 electrode is conditioned with a band-pass filter to isolate sub-band EEG signals (theta: 4–7 Hz, alpha: 8–12 Hz, and beta: 13–35 Hz). The cortico-posture interaction between low-frequency postural fluctuations (< 4 Hz) and high-frequency EEG at C4 is characterized using band-specific PAC. The PAC between postural fluctuations and sub-band EEG across eighteen bins is displayed at the bottom of the plot. The divergence between the observed distribution of phase-amplitude relationships (MI_observed_) is normalized with respect to a reference uniform distribution (MI_shuffled_) across the eighteen bins, resulting in Z_MI_

Phase–amplitude coupling (PAC) analysis was conducted on the phase of postural fluctuations and the amplitude of sub-band EEG signals across all recording electrodes (Fig. 3). The instantaneous phase of the low-frequency postural fluctuation signal was determined using the angle of the analytic signal derived from the Hilbert transform. The phases of these fluctuations were categorized into 18 bins (Δϕ = π/9), and the amplitude of each sub-band EEG (theta (4–7 Hz), alpha (8–12 Hz), and beta (13–35 Hz)) was averaged within each phase bin [32]. Following this, the mean amplitude for all sub-band oscillations was calculated for each of the 18 phase bins, resulting in a band-specific phase–amplitude plot for each experimental trial (see Fig. 3, Bottom). To quantify the divergence of the phase–amplitude plot, the modulation index (MI) was employed, utilizing Kullback–Leibler distance and Shannon entropy [32, 33]. $\:MI=\frac{log\left(N\right)+{\sum\:}_{j=1}^{N}P\left(j\right)log\left|P\left(j\right)\right|}{\text{log}\left(N\right)}$, where P(j) is the amplitude for a given bin j, N is the number of bins (*N* = 18), and log(N) represents the entropy of a uniform distribution.

A shuffled modulation index (MI) was generated by calculating the MI value between the permuted time series of the phase components of postural fluctuations and the amplitude time series of sub-band EEG signals [32]. This shuffling process was repeated 300 times throughout the study. Subsequently, a standardized modulation index (Z_MI_) was computed in relation to the distribution of the shuffled coupling values using the following formula:

$${Z_{MI}} = {{M{I_{ - observed{\rm{ }}}} - {\mu _{M{I_{ - shuffled }}}}} \over {{\sigma _{M{I_ - }shuffled{\rm{ }}}}}}$$

where MI denotes the PAC coupling value, µ denotes the mean, and σ denotes the standard deviation (SD). Data analysis was conducted offline in Matlab R2019a (The Mathworks Inc., Natick, USA).

### Statistical analysis

The postural and standardized PAC variables from the three experimental trials in the full-vision (control) and SV condition were averaged for each subject, respectively. A paired t-test was employed to compare the root mean square (RMS) of postural fluctuation amplitude between control and SV conditions. Multivariate Hotelling’s T-squared statistics were applied to examine the significance of the SV effect on SDA variables, including CT, CD, Ds, Dl, Hs, and Hl. Post-hoc tests were conducted using paired t-tests, with significance determined using Holm’s step-down test to ascertain the level of significant difference. For all post-hoc hypotheses ($\:H={\cap\:}_{i=1}^{m}$), Holm’s test did not reject elementary *H*_*i*_ if *p*_*i*_ ≤ i*0.05/m for ordered unadjusted p values (*p*_*1*_ ≤ … ≤ *p*_*m*_). The type 1 error rate using Holm’s test was exactly 0.05 without the over-correction problem of the Bonferroni test. A paired t-test was used to examine differences in PAC values (or Z_MI_) in the theta, alpha, and beta bands of each EEG channel between the control and SV conditions. This analysis helped identify regions of interest where PAC in different sub-bands was influenced by the SV effect. Subsequently, the paired t-test was used to contrast average Z_MI_ in the regions of interest of each EEG sub-band between the control and SV condition. Pearson’s correlation analysis was conducted to assess the significance of the correlation between normalized differences (ND) in SDA variables of postural fluctuations and ND in Z_MI_ of various sub-bands due to the SV effect (ND = (SV-control)/│control│). All statistical analyses were performed in IBM SPSS Statistics (v19). The level of significance was 0.05.

## Results

### SDA properties of postural fluctuations

The results of paired t-tests showed that the RMS of postural fluctuations was larger in the SV condition than in the control condition (*t*_*32*_ = 8.885, *p* < 0.001). Table 1 summarizes the results of Hotelling’s T-squared statistics comparing the SDA variables of postural fluctuations between the SV and control conditions. The SDA variables differed with and without SV (Wilks’ Λ = 0.367, *p* < 0.001). Post-hoc analysis revealed that intermittent visual feedback resulted in greater CD (*p* < 0.001), Ds (*p* < 0.001), and Hs (*p* < 0.001) than full visual feedback. However, CT, Dl, and Hl did not significantly differ between the control and SV conditions (*p* > 0.05)).

**Table 1:** The means and standard deviations of variables of stabilogram diffusion analysis (SDA) between the control and stroboscopic vision (SV) conditions. (^*^: SV > control, *p* < 0.05; ^***^: SV > control, *p* < 0.001)

SDA Variables	Control	SV	Statistics
CT (second)	1.330 ± 0.389	1.439 ± 0.583	**Λ = 0.367**, ***p*** **< 0.001**
CD (deg^2^)	**20.50 ± 14.86**	**48.78 ± 36.87** ^***^	
Ds (deg^2^/second)	**7.20 ± 5.52**	**15.83 ± 9.37** ^***^	CT: *t*_*32*_ = -1.305, *p* = 0.201; **CD**: ***t***_***32***_**= -5.898**, ***p*** **< 0.001**
Dl (deg^2^/second)	0.186 ± 0.387	0.330 ± 0.670	**Ds**: ***t***_***32***_**= -6.671**, ***p*** **< 0.001**; Dl: *t*_*32*_ = -1.200, *p* = 0.239
Hs (deg^2^/second)	**0.966 ± 0.008**	**0.969 ± 0.007** ^*^	**Hs**: ***t***_***32***_**= -2.614**, ***p*** **= 0.014**; Hl: *t*_*32*_ = -0.201, *p* = 0.842
Hl (deg^2^/second)	0.095 ± 0.076	0.098 ± 0.066	

### PAC between postural fluctuations and EEG

**Fig. 4:**
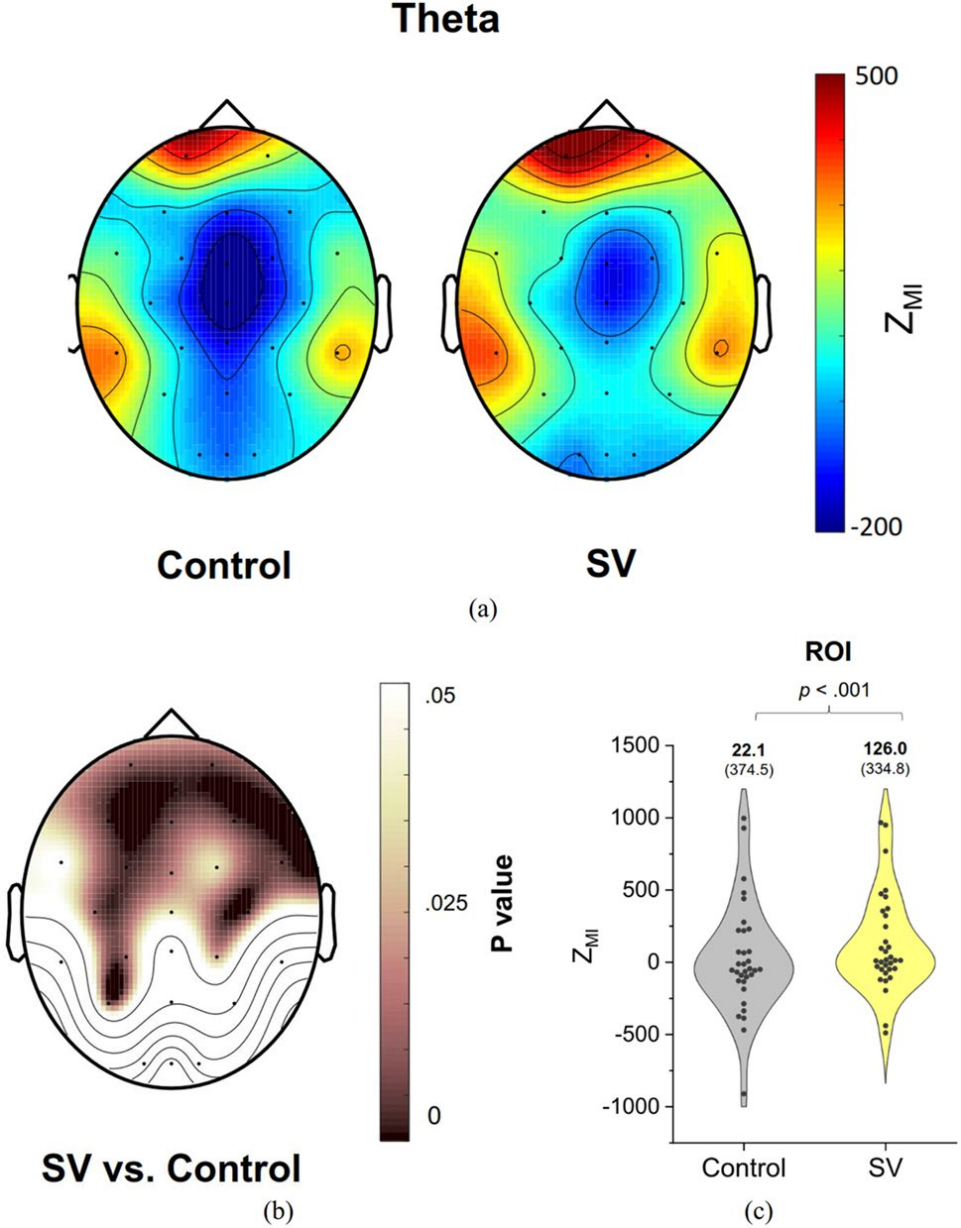
(**A**) Topological distribution of phase–amplitude coupling (PAC) in the theta band (4–7 Hz), involving the phase component of postural fluctuations and band-specific EEG amplitude, for the control and SV conditions. (**B**) Scalp map of p values derived from paired t-tests, reflecting significant differences in Z_MI_ between the control and SV conditions. (**C**) The contrast of pooled Z_MI_ in the regions of interest (ROI), showing significant SV-related differences in theta PAC during stabilometer stance

In terms of Z_MI_, Fig. 4(a) illustrates the population means of the topological distribution of PAC between the phase of postural fluctuations and the amplitude of theta oscillation (4–7 Hz) during stabilometer stance in the control and SV conditions. Figure 4(b) displays the SV-related differences in PAC topological distribution examined by paired t-test with corresponding p-values. The regions of interest are labeled with the topological distribution of the p-values, encompassing several fronto-motor areas (Fp1, Fp2, F3, Fz, F4, F8, FC3, FCz, FC4, FT8, T3, C3, Cz, and C4) and the parietal area (P3, CP3, and P4) (*p* < 0.05). Figure 4(c) presents the results of a paired t-test contrasting the pooled Z_MI_ of theta PAC in these regions of interest between the control and SV conditions. The mean theta PAC in the regions of interest was significantly larger in the SV condition than in the control condition (*t*_32_ = -3.887, *p* < 0.001).

**Fig. 5:**
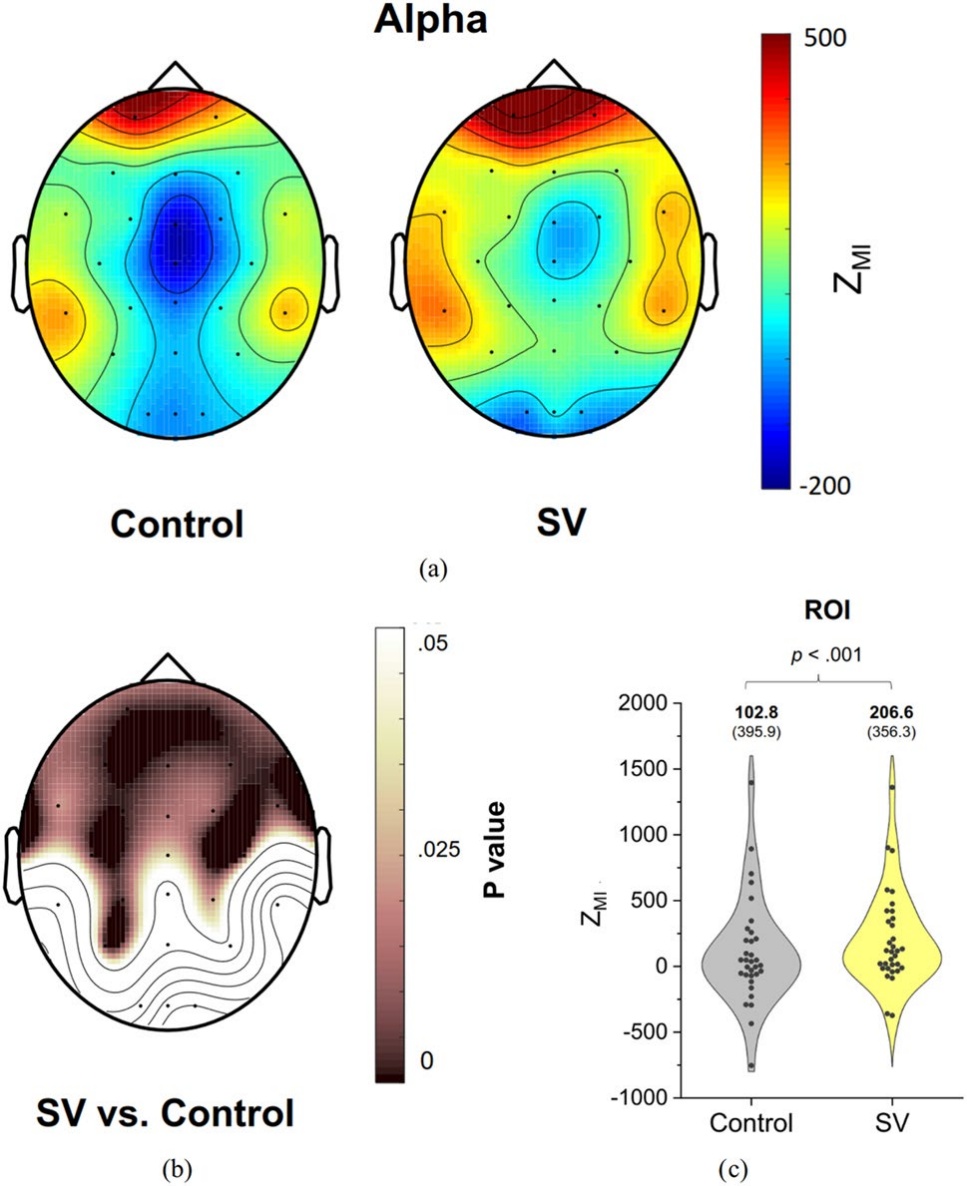
(**A**) Topological distribution of phase–amplitude coupling (PAC) in the alpha band (8–12 Hz), involving the phase component of postural fluctuations and band-specific EEG amplitude, for the control and SV conditions. (**B**) Scalp map of p values derived from paired t-tests, reflecting significant differences in Z_MI_ between the control and SV conditions. (**C**) The contrast of average Z_MI_ in the regions of interest (ROI), showing significant SV-related differences in alpha PAC during stabilometer stance

Figure 5(a) depicts the pooled topological distribution of PAC, or Z_MI_, between the phase of postural fluctuations and the amplitude of alpha oscillations (8–12 Hz) during stabilometer stance under the control and SV conditions. Figure 5(b) highlights the differences in PAC topological distribution between these conditions using paired t-statistics, along with the associated p-values. The regions showing significant SV-related differences in alpha PAC include several anterior cortical areas, such as bilateral fronto-motor regions (Fp1, Fp2, F7, F3, Fz, F4, F8, FT7, FC3, FCz, FC4, FT8, T3, C3, Cz, and C4) and parts of the parietal cortex (P3, CP3, and P3) (*p* < 0.05). Figure 5(c) presents the results of a paired t-test comparing the pooled Z_MI_ of alpha PAC in these regions of interest between the control and SV conditions. The findings indicate that mean alpha PAC in these regions was significantly higher in the SV condition than in the control condition (*t*_32_ = -4.089, *p* < 0.001).

**Fig. 6:**
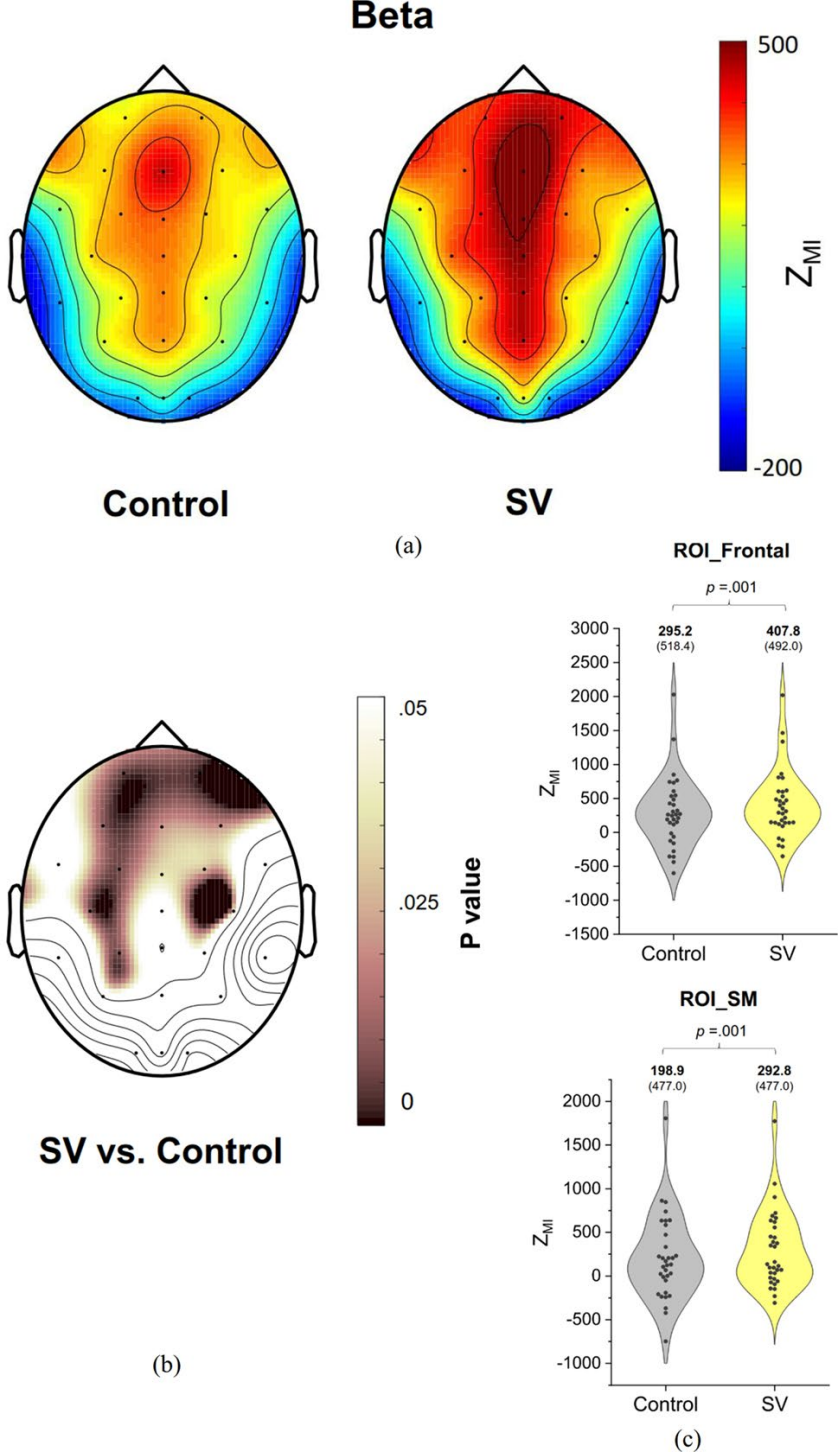
(**A**) Topological distribution of phase–amplitude coupling (PAC) in the beta band (13–35 Hz), involving the phase component of postural fluctuations and band-specific EEG amplitude, for the control and SV conditions. (**B**) Scalp map of p values derived from paired t-tests, reflecting significant differences in Z_MI_ between the control and SV conditions. (**C**) The contrast of average Z_MI_ in the regions of interest (ROI), frontal and sensorimotor (SM) areas, showing significant SV-related differences in beta PAC during stabilometer stance

Figure 6(a) illustrates the pooled topological distribution of PAC, or Z_MI_, between the phase of postural fluctuations and the amplitude of beta oscillations (13–35 Hz) during stabilometer stance in both control and SV conditions. Figure 6(b) presents the paired t-statistics results, indicating the differences in PAC topological distribution between these conditions, along with the corresponding p values. The regions showing significant SV-related differences in beta PAC include several anterior cortical areas, such as the frontal (Fp1, Fp2, F3, Fz, F4, F8) and sensorimotor cortices (FC3, FCz, FC4, T3, C3, C4, and CP3) (*p* < 0.05). The results of a paired t-test indicated that the SV condition exhibited a greater pooled Z_MI_ in the frontal (*t*_32_ = -3.614, *p* = 0.001) and sensorimotor areas (*t*_32_ = -3.676, *p* = 0.001) compared to the control condition (Fig. 6(c)).

**Fig. 7:**
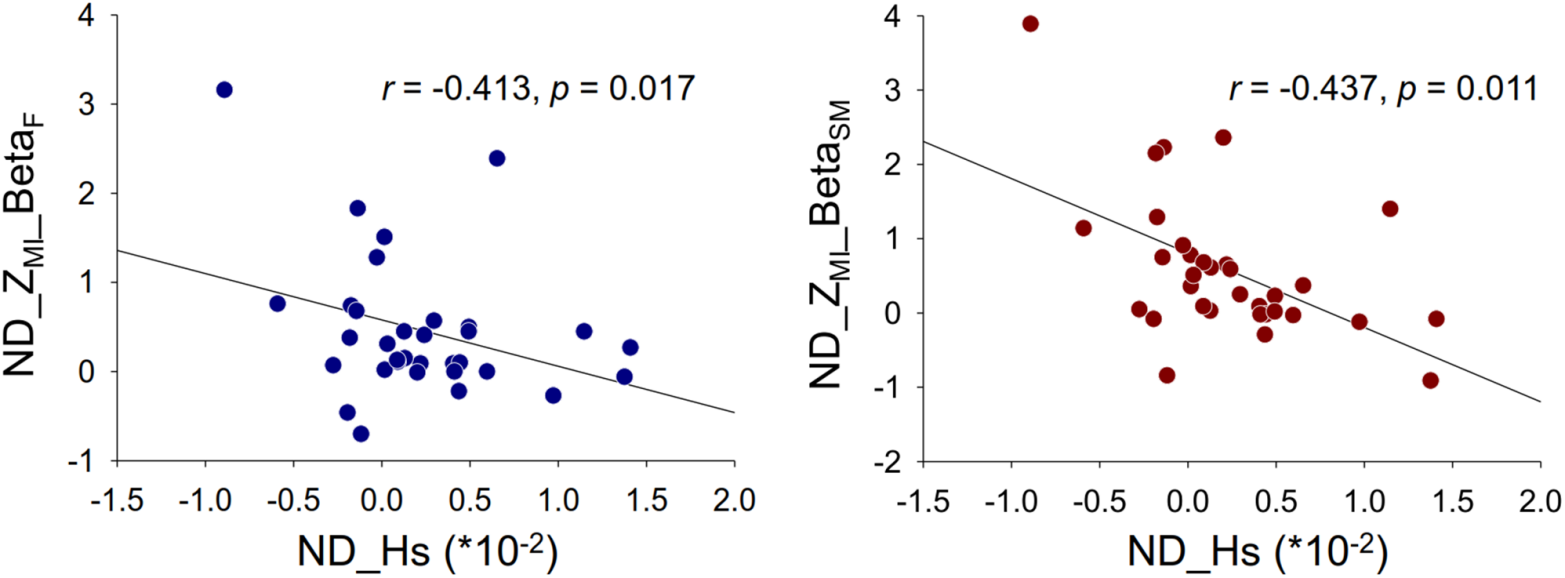
Significant Pearson correlation between normalized differences in stabilogram diffusion variable and normalized differences in phase-amplitude coupling in the beta band. The left plot is correlational plot of normalized differences in Hs (ND_Hs) and Z_MI_ of the frontal area in the beta band (ND_Z_MI__Beta_F_). The right plot is correlational plot of normalized differences in Hs (ND_Hs) and Z_MI_ of the sensorimotor area in the beta band (ND_Z_MI__Beta_SM_)

Pearson’s correlation was performed between normalized differences (ND) in SV-dependent SDA variables (CD, Ds, and Hs) and pooled Z_MI_ in the regions of interest of different sub-bands. There was no significant correlation between SV-dependent SDA variables and the pooled Z_MI_ of the regions of interest (*p* = 0.137 to *p* = 0.978), except for correlations between ND in Hs and pooled Z_MI_ in the frontal and sensorimotor areas in the beta band (*p* < 0.05) (Fig. 7).

## Discussion

This study demonstrated that intermittent visual feedback introduced additional postural challenges for older adults during the stabilometer stance, emphasizing the reliance on an open-loop control scheme. The alteration in postural control strategy capitalizes on the reorganization of cortico-posture interactions, which is evidenced by variations in PAC between postural fluctuations and scalp EEG across the theta, alpha, and beta bands related to SV. Notably, the increase in the open-loop gain of the postural system associated with SV was found to be negatively correlated with changes in beta power of PAC within the frontal and sensorimotor regions.

### Enhanced open-loop control of postural system with intermittent visual feedback

Due to larger sway, intermittent visual feedback posed a greater postural challenge for older adults during stabilometer stance. Using SDA, it was found that stochastic properties of postural fluctuations varied with SV. SDA categorizes these fluctuations into short-term and long-term control mechanisms, separated by a critical transition point [29, 30]. Short-term fluctuations (Hs > 0.5) are persistent and regulated by open-loop control to mitigate rapid threats, while long-term fluctuations (Hl < 0.5) are stationary and exhibit anti-persistence under closed-loop control [29, 30]. Increased displacement at short timescales triggers the closed-loop process to maintain postural consistency with feedback adjustments once critical displacement (CD) is exceeded.

Within the context of SDA, intermittent visual feedback increased CD (Table 1), indicating that the postural system’s feedback control was less sensitive to postural variances, thereby favoring open-loop control during stabilometer stance until greater postural errors occurred. Additionally, although visual feedback was partially blocked, the overall feedback gain of the postural system in the older adults was not significantly affected by SV, as evidenced by insignificant changes in Hl and Ds (Table 1). This observation aligns with previous findings that the long-term diffusion exponent in older adults is minimally affected by the removal of visual information [31, 34]. Along with an insignificant delay in feedback time (CT), the feedback efficacy of the postural system after partial visual occlusion is largely compensated by non-visual channels.

Notably, the open-loop gain (Ds and Hs) of the postural system significantly increased with SV (Table 1). This increased gain in the open-loop process is beneficial for training older adults to respond rapidly to sudden balance losses or unexpected perturbations. From a rehabilitation perspective, improving open-loop gain can aid recovery from postural threats in everyday activities by enhancing anticipatory control and utilizing well-practiced pre-programmed movements [35]. Evidence suggests that the increase in open-loop gain is achieved through enhanced reflex responses or increased ankle joint stiffness via coactivation of antagonist muscle pairs [29, 36, 37]. However, the exact central mechanisms behind SV-induced increases in open-loop gain remain debated.

### Variations in brain-posture interaction with intermittent visual feedback

The SV-induced increases in PAC between force fluctuations and EEG in the theta and alpha bands were predominantly observed in the fronto-central areas (Figs. 3 and 4). However, SV-related changes in PAC in the theta/alpha bands did not significantly correlate with SV-related changes in critical time displacement and open-loop gain (Ds and Hs) (*p* > 0.05). Therefore, theta and alpha PAC did not contribute to the shift in postural control toward a more feedforward approach. For motor responses, error signatures in the theta band may be widespread across cortical areas involved in movement generation, beyond just the mid-frontal region [38, 39]. Since theta activity in the fronto-central area is linked to postural error monitoring, the SV-induced increase in theta PAC may reflect cortico-posture connectivity with a higher task demand when processing visual error becomes more challenging due to intermittent visual feedback.

Similar SV-induced PAC potentiation in the alpha band was also noted in the fronto-central area. Opposing hypotheses regarding alpha oscillations in postural tasks include the idling hypothesis [40], which views alpha power as a marker of cortical inactivity, and the individual alpha peak frequency (iAPF) hypothesis [41], which sees it as regulating thalamo-cortical information transmission. According to the iAPF framework, increased balance task demands are associated with higher alpha power in the frontal and fronto-central regions [42, 43]. In the context of balance control, the iAPF hypothesis is better suited for interpreting the covariant changes in theta/alpha PAC with intermittent visual feedback in this study. Collectively, for older adults, the SV-induced increase in alpha PAC reflected the heightened task demands to stabilize postural response on the stabilometer with intermittent visual feedback. This argument is supported by the positive correlation between the RMS of postural fluctuations in the SV condition and the SV-related increase in alpha PAC (ND_Z_MI__Alpha) (*r* = 0.459, *p* = 0.007).

SV also enhanced beta PAC in the frontal and sensorimotor areas (Fig. 6). Conceptually, this increase in beta PAC aligns with previous findings of elevated beta-band power in the parietal and central regions of the brain during challenging postural tasks involving various sensory perturbations [44] or rapid mechanical perturbations [45]. Enhanced beta rhythms in these regions reflect heightened cortical engagement, integrating sensory processing, motor execution, and executive functions to effectively manage postural difficulty. Specifically, in the sensorimotor cortex, beta rhythms facilitate large-scale neural communication within the corticospinal tract, aiding in the coactivation of antagonist muscle pairs. This coactivation improves fine motor control and torque stability in the knee and ankle joints [46, 47]. The increase in beta PAC in the sensorimotor area appears to reinforce open-loop control during stabilometer stance with SV, underlying the enhanced ankle joint stiffness achieved through antagonist coactivation [29, 36, 37].

However, we observed a negative correlation between ND in Hs and ND in pooled Z_MI_ in the frontal and sensorimotor areas (Fig. 7), which questions the proposed role of beta PAC in top-down processes for reinforcing open-loop control. The observed negative correlation suggests that a smaller increase in beta PAC corresponded with a greater SV-induced increase in open-loop gain, despite an overall enhancement of beta PAC in older adults following partial visual occlusion. It has also been reported that beta oscillations in the frontal [48, 49] and sensorimotor [49–51] adjusts motor planning in response to varying levels of predictability and control over movements. Within this notion, an alternative interpretation is that beta PAC in these areas may link to the degree of uncertainty experienced by older adults who rely on visual input for postural maintenance when visual feedback is impaired. In a target-matching experiment with visual guidance, Tan et al. (2016) found that reduced beta amplitude in the sensorimotor area was linked to greater exploratory adjustments to process motor uncertainty following visual perturbation [52]. Additionally, Palmer et al. (2019) reported an inverse relationship between sensorimotor beta power and uncertainty, or sensory prediction errors, during a visuomotor adaptation paradigm [53]. In this context, older adults who exhibited a smaller increase in beta PAC in the sensorimotor and frontal areas demonstrated greater movement uncertainty during stabilometer stance with SV. This uncertainty prompted a reduction in feedback strategies for postural regulation, resulting in a higher open-loop gain (ND_Hs) in destabilizing situations. Conversely, some older adults with higher beta PAC were more confident in their movement anticipation, leading to minimal changes in ND_Hs or a reduced reliance on open-loop control with SV (negative ND_Hs) [54]. In summary, SV introduced visual feedback uncertainty prompting a transition in postural sway towards an open-loop process for the older adults. The shift in postural strategy were linked to the regulation of postural fluctuation phases by beta oscillations in the frontal and sensorimotor areas.

## Conclusion

This study highlights a significant change in postural regulation and variations in cortico-posture connectivity through the application of stroboscopic vision in older adults maintaining an unstable stance, as revealed by SDA and PAC analysis. The use of stroboscopic glasses during an unsteady position increases the challenge of balance, suggesting that older adults employ a more efficient open-loop control mechanism to counteract stance instability. Additionally, intermittent visual feedback influences the cross-frequency coupling between the phase of postural fluctuations and the amplitude of scalp EEG. An increase in theta/alpha PAC observed in the fronto-central regions indicates the central postural control’s adaptation to the heightened balance challenges associated with stroboscopic vision. Furthermore, the shift toward open-loop control in response to stroboscopic vision is functionally modulated by beta PAC in both frontal and sensorimotor regions. These findings suggest the potential benefits of integrating stroboscopic vision with stabilometer training to enhance postural response agility and promote anticipatory control strategies, thereby aiding in fall prevention among older adults.

## Data Availability

The sample datasets generated and analyzed in the study are available from the corresponding author upon reasonable request.

## Abbreviations

CD: Critical Displacement
CT: Critical Time
Dl: Long-term Effective Diffusion Coefficients
Ds: Short-term Effective Diffusion Coefficients
EEG: Electroencephalography
EOG: Electrooculography
Hl: Long-term scaling exponent
Hs: Short-term scaling exponent
iAPF: Individual Alpha Peak Frequency
MI: Modulation Index
ND: Normalized Differences
PAC: Phase-Amplitude Coupling
RMS: Root Mean Square
SDA: Stabilogram Diffusion Analysis
SV: Stroboscopic Vision
Z_MI_: Modulation Index
